# Comparison between transthoracic echocardiography and transoesophageal echocardiography in the diagnosis of acute aortic dissection from an emergency perspective. A systematic review and meta-analysis

**DOI:** 10.3389/fcvm.2023.1283703

**Published:** 2024-01-10

**Authors:** Hany A. Zaki, Bilal Albaroudi, Eman E. Shaban, Baha Hamdi Alkahlout, Yavuz Yigit, Wael Elnabawy, Kaleem Basharat, Nood Dhafi Almarri, Aftab Mohammad Azad

**Affiliations:** ^1^ Department of Emergency Medicine, Hamad Medical Corporation, Doha, Qatar; ^2^ Department of Cardiology, Al Jufairi Diagnosis and Treatment, MOH, Doha, Qatar; ^3^ Centre for Neuroscience, Blizard Institute, Queen Mary University, London, UK; ^4^ Hamad Medical Corporation, Collège of Medicine QU and Weil Cornell Medical College, Doha, Qatar

**Keywords:** aortic dissection, emergency medicine, meta-analysis, point-of-care systems, ultrasound, echocardiography, sensitivity, specificity

## Abstract

**Background:**

Acute aortic dissection (AAD) is a life-threatening medical condition with high early fatality. Therefore, a prompt and precise diagnosis, which can be achieved through invasive and non-invasive techniques is vital. Echocardiography, unlike MRI and CT, is accessible in emergency units and bedside-compatible. The recommended echocardiographic techniques for AAD are transthoracic and transoesophageal echocardiography (TTE and TOE). Therefore, our review compares their diagnostic roles in AAD.

**Methods:**

Studies relevant to our topic were attained through a database search and manual scrutiny of references lists of articles obtained from the electronic databases. The Quality Assessment of Diagnostic Accuracy Studies tool (QUADAS-2) has been used for quality assessment. All quantitative analyses were performed using either STATA 16 or Comprehensive Meta-Analyst software.

**Results:**

The search strategy yielded 1,798 articles, of which only 11 were eligible for inclusion. Our subgroup analysis showed that conventional TTE had a sensitivity and specificity of 85.35% and 84.51% for the diagnosis of Stanford type A AAD and was 45.89% sensitive and 87.05% specific for the diagnosis of type B AAD. However, the subgroup analysis shows that contrast-enhancement of TTE results in a sensitivity and specificity of 93.30% and 97.60% for diagnosis of type A AAD, and 83.60% and 94.50% for diagnosis of type B AAD, respectively. On the other hand, conventional TOE was 93.64% sensitive and 95.50% specific for the diagnosis of type A AAD, 99.80% sensitive and 99.87% specific for the diagnosis of type B AAD. Moreover, our analyses show that TTE has pooled false negative and positive rates of 28.6% and 18.6%, while TOE has shown false negative and positive rates of 2.4% and 4.3%, respectively.

**Conclusion:**

TOE is the more favorable diagnostic tool for AAD diagnosis than TTE. However, it cannot be used as a stand-alone diagnostic tool since misdiagnosis cases are being reported. Contrast-enhanced TTE can also diagnose AAD since it provides similar results to conventional TOE.

## Introduction

Acute aortic dissection (AAD) is the most prevalent life-threatening condition that impairs the aorta, with a mortality rate that ranges from 1% to 1.4% per hour when untreated (1, 2). The death rate is generally substantial in type A dissection (involving the ascending aorta), with reports suggesting nearly 58% mortality rate in patients without treatment and 26% in those with surgical therapy (3). On the other hand, a lower mortality rate is observed in the type B dissection, with reports showing mortality rates of about 31% in patients who underwent surgery and 11% for those medically treated (3). Given these high mortality rates, accurate and early diagnosis of AAD is essential in reducing morbidity and mortality in these patients.

In the early 1960s, aortography was considered the standard tool for diagnosing AD; however, recent technological advancements have led to the use of noninvasive imaging modalities such as echocardiography, magnetic resonance imaging (MRI), and computed tomography (CT). The European Society of Cardiology and the European Association of echocardiography have suggested that transthoracic echocardiography (TTE), which involves the placement of the ultrasound transducer on the chest, be used as the first-line imaging test for patients with suspected AAD especially when the aortic dissection risk score (ADD-RS) (score ranges of 0 to 3, of which 0 is classified as low risk, 1 moderate risk and 2–3 is high risk) shows a low probability for AAD (4, 5). However, TTE is restricted in patients exhibiting abnormal chest wall configuration, obesity, pulmonary emphysema, and mechanically ventilated patients (4). Therefore, transoesophageal echocardiography (TOE), which involves inserting the transducer in the esophagus, has been used to overcome these limitations (4, 6). Previous research studies have shown that TOE has almost similar sensitivity and specificity as CT and MRI; however, due to its lower availability compared to CT and patient discomfort, it is only used as the first-line imaging test in about 30%–40% of the AAD cases (3, 7, 8).

To the best of our knowledge, there is no systematic review comparing TTE to TOE regarding the diagnosis of AAD. Therefore, the current review will compare the diagnostic performance of TOE and TTE in diagnosing AAD and form a basis for emergency medicine care.

## Methodology

### Protocol & registration

This systematic review and meta-analysis was conducted in accordance with the Preferred Reporting Items for Systematic Reviews and Meta-Analyses (PRISMA) 2020 guiding principles and protocol registered on PROSPERO article (CRD42023404905)**.**

### Eligibility criteria

Before any study was included in the current review, the following criteria had to be met;

1. Studies designed as either observational (prospective, retrospective, or cohort) or randomized trials published in English. This criterion was fundamental to our analysis since we did not want to have direct translations of scientific terms that could undermine our research.
2. Studies that evaluated any classification of AAD (either type A or type B in the Stanford classification or type I, II, or III in the DeBakey classification). Type A dissection is defined as dissection proximal to the brachiocephalic artery and involves the ascending aorta, while type B AD originates from the left subclavian artery and involves only the descending aorta. On the other hand, type I AD originates in the ascending aorta to the aortic arch, type II originates and is limited to the ascending aorta, and type III starts in the descending aorta and extends distally above or below the diaphragm.
3. Studies that evaluated either TOE or TTE in the AAD diagnosis.

On the other hand, the reviewers ensured that studies that met the following criteria were excluded from the current review;

1. Studies designed as either abstracts without full articles, systematic reviews, case reports or series, diagnostic algorithms, or letters to the editor.
2. Studies that only evaluated other imaging techniques (CT and MRI) and aortography in diagnosing AAD.
3. Studies that only included patients with Chronic or Asymptomatic AD.
4. Studies with insufficient sample size (less than 30 participants). This criterion was used in this review to help improve the statistical power of our analyses.
5. Studies that evaluated patients with the acute aortic syndrome or AD in general but did not distinguish the data for AAD.

### Literature search

Five electronic databases—Embase, PubMed, ScienceDirect, Scopus, and Google scholar—were scoured for scientific journals published in English. The reviewers created a search strategy that involved combining scientific terms using the Boolean expressions “AND” and “OR”. The strategy employed was as follows; (“Transoesophageal echocardiography” OR “transesophageal echocardiography” OR “TOE” OR “TEE”) AND (“transthoracic echocardiography” OR “TTE”) AND (“Acute aortic dissection” OR “acute Type A aortic dissection” OR “acute Type B aortic dissecting”). Additional studies were obtained by going through the reference lists of relevant studies from the databases mentioned earlier. To avoid undermining our scientific research, grey literature, and close duplicates were not retrieved.

### Quality assessment

The current study was designed as a diagnostic review; therefore, the Quality Assessment of Diagnostic Accuracy Studies tool (QUADAS-2) in the Review Manager software (RevMan 5.4.1) was employed to assess the risk of bias. This tool usually consists of the Assessment of bias category, subdivided into four domains (selection, index test, reference standard, and flow and timing) and applicability concerns category, which consists of three fields, including patient selection, index test, and reference standard. In the quality assessment, low risk of bias was assigned a green color, while high risk and unclear risk of bias were assigned red and yellow, respectively.

### Data extraction and definitions

The data extraction process was carried out by two reviewers who later compiled the data in a tabular form. The data retrieved was as follows; Author ID (first author's surname and the publishment year), patient characteristics (patients enrolled and patients with AAD in the final diagnosis), study design, study location, and reference test, and the main outcomes. The primary outcome of the current review was to compare the sensitivity and specificity of TTE to TOE, while the secondary outcome was to compare misdiagnosis between the two techniques. All the discrepancies in the collected data were initially resolved through interactive discussion between the two reviewers and if a consensus could not be reached a third reviewer was consulted.

According to accepted conventions, AAD represents patients with the sudden onset of symptoms within 48 h, while subacute represents patients with symptoms occurring for at least two weeks. On the other hand, chronic AD represents patients whose symptoms have occurred for more than two weeks. However, for this study, we considered AAD to be patients with symptoms occurring between 1 and 14 days.

### Data synthesis

The diagnostic accuracy analysis was done using STATA 16, while the rate of misdiagnosis was carried out using Comprehensive Meta-analyst software. In these analyses, we employed the randoms effect model to counter the expected heterogeneity, calculated using the I^2^ statistics. The heterogeneity values between 0%–40%, 41%–60%, and 61%–100% were considered low, moderate, and substantial, respectively. All the results related to diagnostic accuracy (specificity and sensitivity) were presented in the form of forest plots. To further enhance our analysis, we subdivided the outcomes based on the type of AAD. The Stanford classification was used, of which the DeBarkley classifications I and II were considered type A AAD, and classification III was considered type B AAD. On the other hand, the results of misdiagnosis, including false negatives and false positives, were recorded in a tabular form. In all these analyses, a 95% confidence interval was applied.

## Results

### Study selection

**Figure 1:**
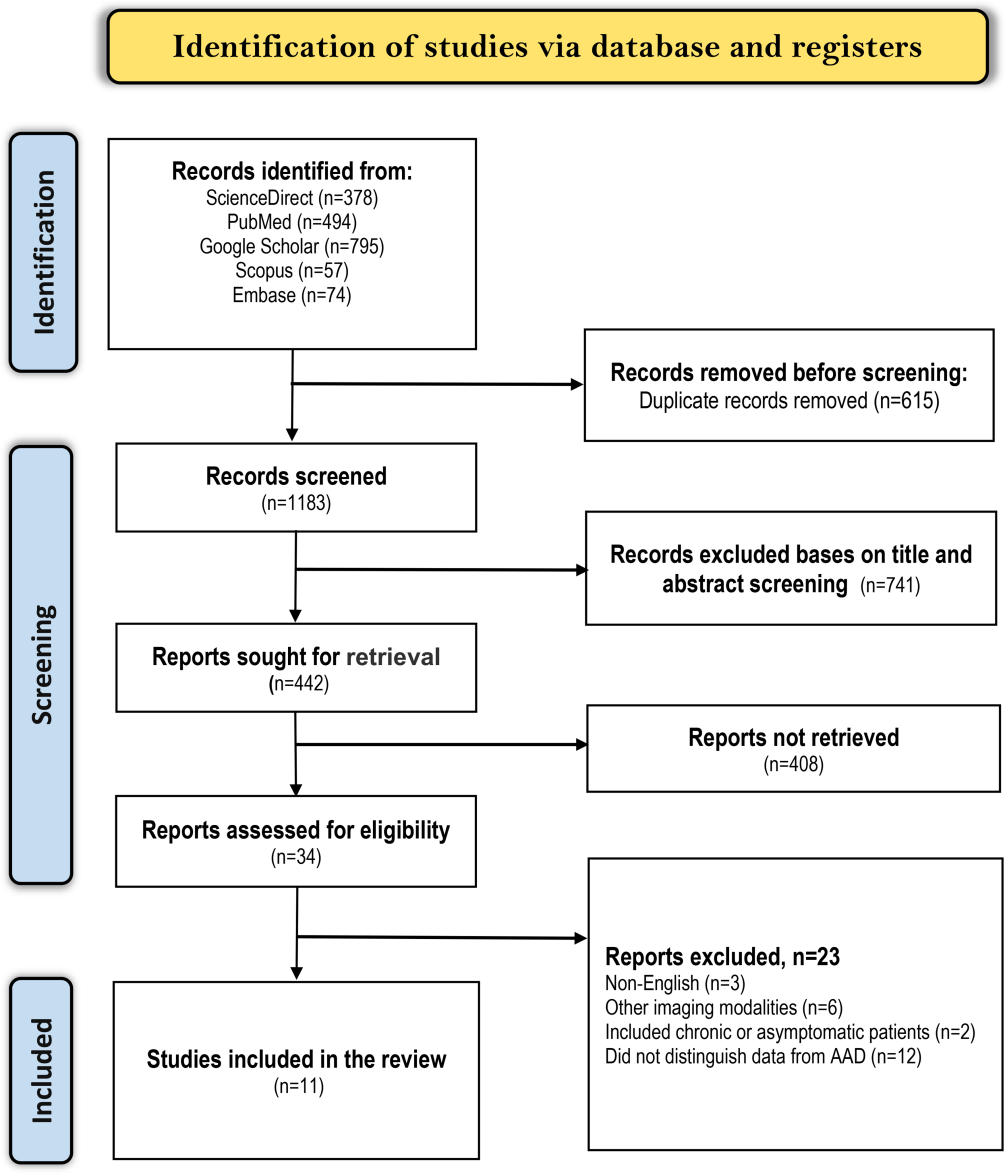
PRISMA flow diagram for study selection.

We applied the search strategy mentioned earlier in the five electronic databases and attained 1,798 articles related to our topic. The articles were then screened for duplicates, of which 815 close (articles with nearly identical content i.e., multiple versions of the same study with minor differences) or exact (articles with identical content) duplicates were excluded. Afterward, the remaining 983 articles were screened by looking at their titles and abstracts, of which 896 were excluded. Out of the 87 remaining articles, we did not retrieve 44 because they were either abstracts without full evidence, letters to the editor, Guidelines, case reports, systematic reviews, or ongoing trials. Finally, 11 articles met the criteria for inclusion while the other 32 articles were excluded as follows; 10 evaluated other imaging techniques in AAD diagnosis, 8 were published in different languages, 2 only included patients with chronic or asymptomatic AD, and 12 included patients with acute aortic syndrome or AD in general but did not distinguish data for AAD. A summarized literature selection criterion is shown in the PRISMA diagram below (Figure 1).

### Summary of study characteristics

Out of the 11 studies, 8 were carried out in single centers (9–16), while the other 3 were conducted in multiple centers (17–19). In these studies, a total of 3,046 patients suspected of AAD were enrolled, of which a majority were men (65% (1995/3,046). Out of the 2,418 patients suspected of AAD in 10 studies, AAD was confirmed in 775 (32%) patients during the final diagnosis, of which type A AAD was confirmed in 661 patient and type B AAD was confirmed in 114 patients (Table 1). The Study by Moore and colleagues (19) did not provide the data for patients with AAD in the final diagnosis; therefore, it was not used to calculated the ratio of patients with AAD in the final diagnosis.

**Table 1:** Summary of study characteristics.

Author ID	Study design	Location	Patient characteristics						Index test	Device used	Reference test	Main outcomes
			Patients enrolled			Patients with AAD in final diagnosis						
			Sample (*n*)	M/F	Mean age (years)	Total (*n*)	Type A (*n*)	Type B (*n*)				
Cecconi et al. (11)	Retrospective study	Italy	270	191/79	66 ± 15	62	62	–	TTE	Sonos 5,500 ultrasound system with an S3 probe and harmonic imaging.	Surgical inspection, autopsy, CT, or MRI.	Overall, TTE was 90% sensitive and 96% specific for the diagnosis of CAAAD. Of the 9 false negatives recorded in the TTE studies, 6 were patients with CAAAD.
Nazerian et al. (12)	Prospective single-center cohort study	Italy	281	155/126	NR	57	50	7	TTE	MyLab30 Gold and HD7 with 2–5 MHz phased array probe.	TOE and CTA.	The presence of at least one sign (indirect or direct) in the TTE studies had a sensitivity and specificity of 88% and 56% for the diagnosis of TAAAD.
Sobczyk et al. (13)	Retrospective single-center study	Poland	178	131/47	58.69 ± 12.61	171	171	–	TTE	Vivid I (GE) or CX 50 (Phillips) ultrasound system with an S3 3.5–5 MHz transducer.	CT and actual surgical findings	TTE detected the initial dissection flap in the ascending aorta in 159 patients, while the intraoperative inspection detected TAAAD in 171 patients.
Asouhidou et al. (14)	Retrospective single-center study	Greece	49	41/8	54.8 ± −9.2	49	43	6	TTE	NR	Chest x-ray, CT, and Coronary angiography	Of the 24 patients who underwent TTE studies, 50% (12/24) were true positives, and 50% (12/24) were false negatives
Evangelista et al. (15)	Retrospective single-center study	Spain	128	92/36	61 (49–74)	76	45	31	TTE and TOE	GE Vivid 1 or Vivid 7 with a 1.5–4.3 MHZ transducer using harmonic imaging in TTE studies and multiplanar probe with a 2.9–8 MHz transducer using harmonic imaging in TOE studies	CT, MRI, and surgical findings	Overall specificity and sensitivity for AAD diagnosis using TTE were 71.2% and 73.7%, respectively, while for TOE were 94.2% and 97.3%, respectively. TTE showed a sensitivity and specificity of 82.2% and 89.2% for diagnosing Ascending aorta dissection, 79.1% and 88.2% for diagnosing aortic arch dissection, and 56.2% and 81.8% for the diagnosis of descending aorta dissection. TOE showed a sensitivity and specificity of 95.6% and 96.4% for the diagnosis of Ascending aorta dissection, 90.7% and 98.8% for the diagnosis of aortic arch dissection, and 100% for the diagnosis of descending aorta dissection.
Nienaber et al. (16)	Prospective single-center study	Germany	53	35/18	52 ± 15	31	20	11	TTE and TOE	2.25–3.5 MHz transducers attached to V3400 R CV60, HP77065 or HP Sonos 1,000 sector scanner	MRI and surgical findings.	TOE had a sensitivity of 100% for AD diagnosis, while TTE was 82.7% sensitive. TOE had a false-positive result in 7 patients, thus resulting in a specificity of 68.2% TTE showed a sensitivity and specificity of 85% and 78.1% for the diagnosis of type A AD, while TOE had a sensitivity and specificity of 100% and 78.8%, respectively. TTE showed a sensitivity and specificity of 55.5% and 100% for the diagnosis of type B AD while TOE had a sensitivity and specificity of 90.9% and 97.6%, respectively.
Agricola et al. (17)	Single center retrospective study	Italy	66	44/22	67 ± 13.3	22	9	13	TOE	IE33 Philips with 2–7 MHz Omni III probe.	Gated-CT angiography	The standard TOE diagnosed aortic dissection with a sensitivity and specificity of 91% and 95%, respectively.
Keren et al. (18)	A single center retrospective study.	United States	112	80/32	60.6 ± 16.8	49	30	19	TOE	Sonos 1,000 and 1,500	CT, Angiography, MRI, surgery, and autopsy.	The specificity and sensitivity of TOE in identifying AD was 90% (79–100) and 98% (95–100) for type A and 100% and 99% (97%–100%) for type B. The TOE findings were false negative in 3 patients (2 with type A and 1 with type B).
Hui et al. (19)	Retrospective study of two centers	China	442	272/170	NR	146	146	–	TTE	NR	Surgical findings	When any signs of TTE were considered for the diagnosis of TAAAD, a 97% sensitivity and 78% specificity was recorded.
Nazerian et al. (20)	A multicenter prospective study	Brazil, Germany, Italy, and Switzerland	839	540/299	62 ± 16.7	112	85	27	TTE	The following multiprobe machines with a 2–5 MHz phased array probe were used: two MyLab 5, two MyLab30 Gold, two MyLab alpha (Esaote, Genova, Italy), one HD7 (Koninklijke Philips, Amsterdam, Netherlands), three Vivid S5, and one Vivid S6 (GE Healthcare, Wauwatosa, WI, USA)	CTA, TOE, MRA, surgery and autopsy	The sensitivity and specificity of TTE associated with any sign was 96.5% and 70.2% for diagnosis of type A AAD.
Moore et al. (7)	A multicenter retrospective study.	United States, Italy, Japan, Germany, Spain, and Israel	628	414/214	63.6 ± 13.7	NR	NR	NR	TOE	NR	CT, MRI, and angiography.	TOE had an overall sensitivity of 88% for AD diagnosis (90% for Type A and 80% for type B). The TOE findings were false negative in 23 patients (14 with type A and 7 with type B).

AAD, acute aortic dissection; TTE, transthoracic echocardiography; TOE, transoesophageal echocardiography; CT, computed tomography; MRI, magnetic resonance imaging; TAAAD; type A acute aortic dissection; CAAAD; classic type A acute aortic dissection; NR, not reported; MRA, magnetic resonance angiography.

### Quality assessment results

**Figure 2:**
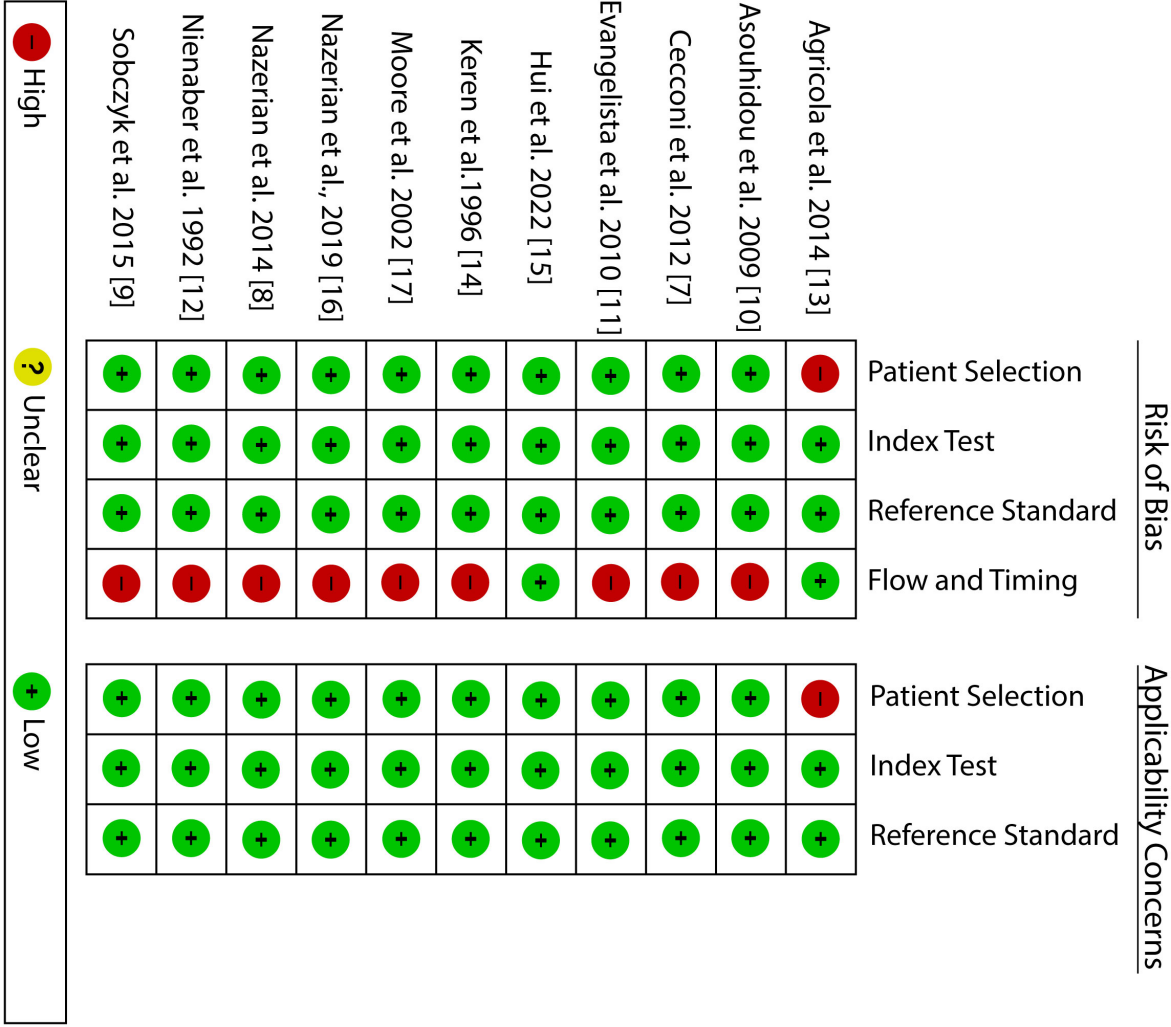
QUADAS-2 bias assessment summary.

The risk of bias summary is presented in Figure 2 below. From the summary, it is evident that all the studies satisfy at least four of the seven assessment criteria, meaning that they had a low risk of bias. Considering the patient selection criteria all the studies had a low risk of bias and low concern except for one study which used a non-consecutive patient selection and included about 50% with an already established suspicion for AAD. The index and reference test assessment criteria also showed low risk and low concern; however, it is worth noting that in most studies it was unclear whether the reference test was conducted without knowing the results of the index test. On the other hand, with regard to flow and timing, all studies except two showed a high risk of bias. The high risk of bias was because these studies evaluated more than one reference test.

### TTE diagnostic accuracy

**Figure 3:**
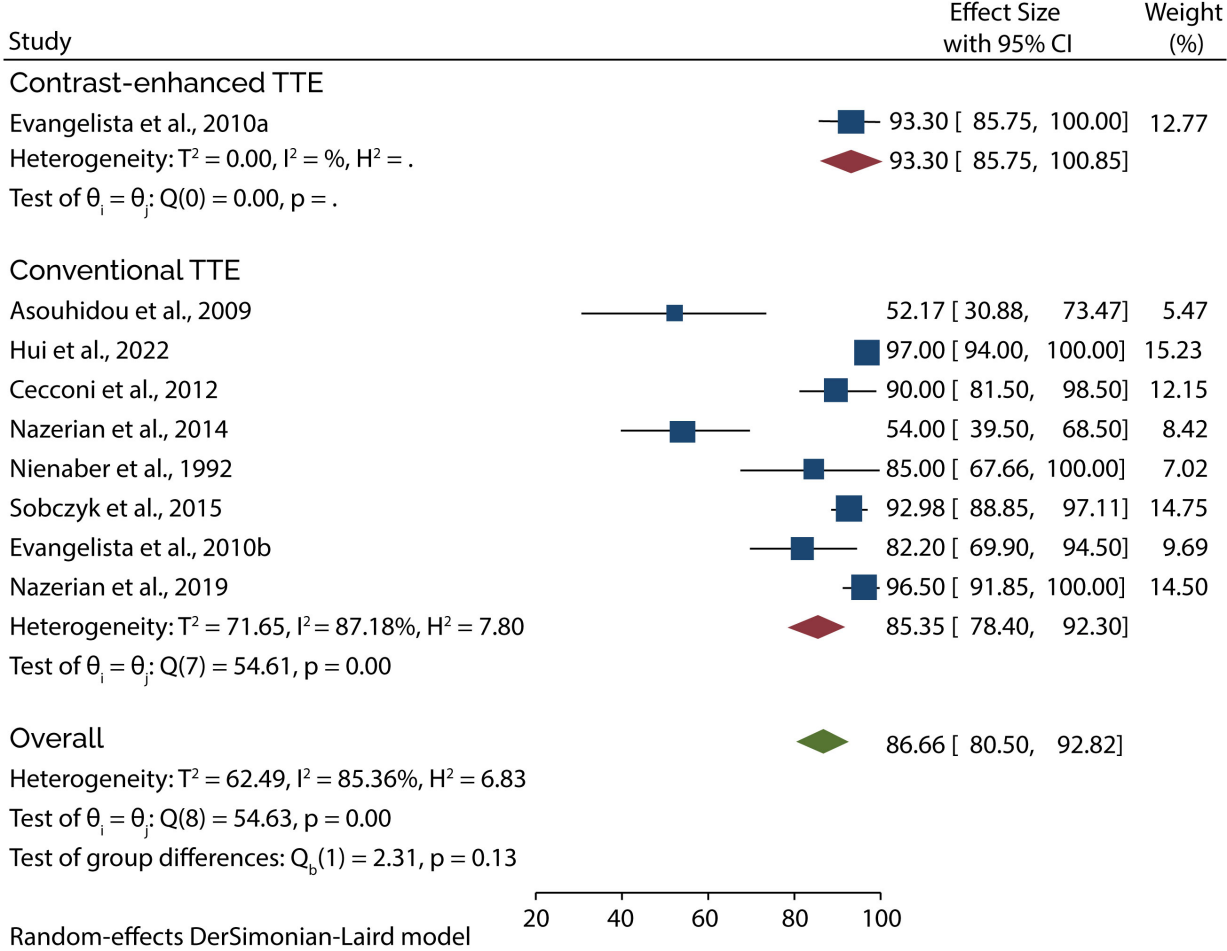
Forest plot of pooled sensitivity of TTE in diagnosing type A AAD.

**Figure 4:**
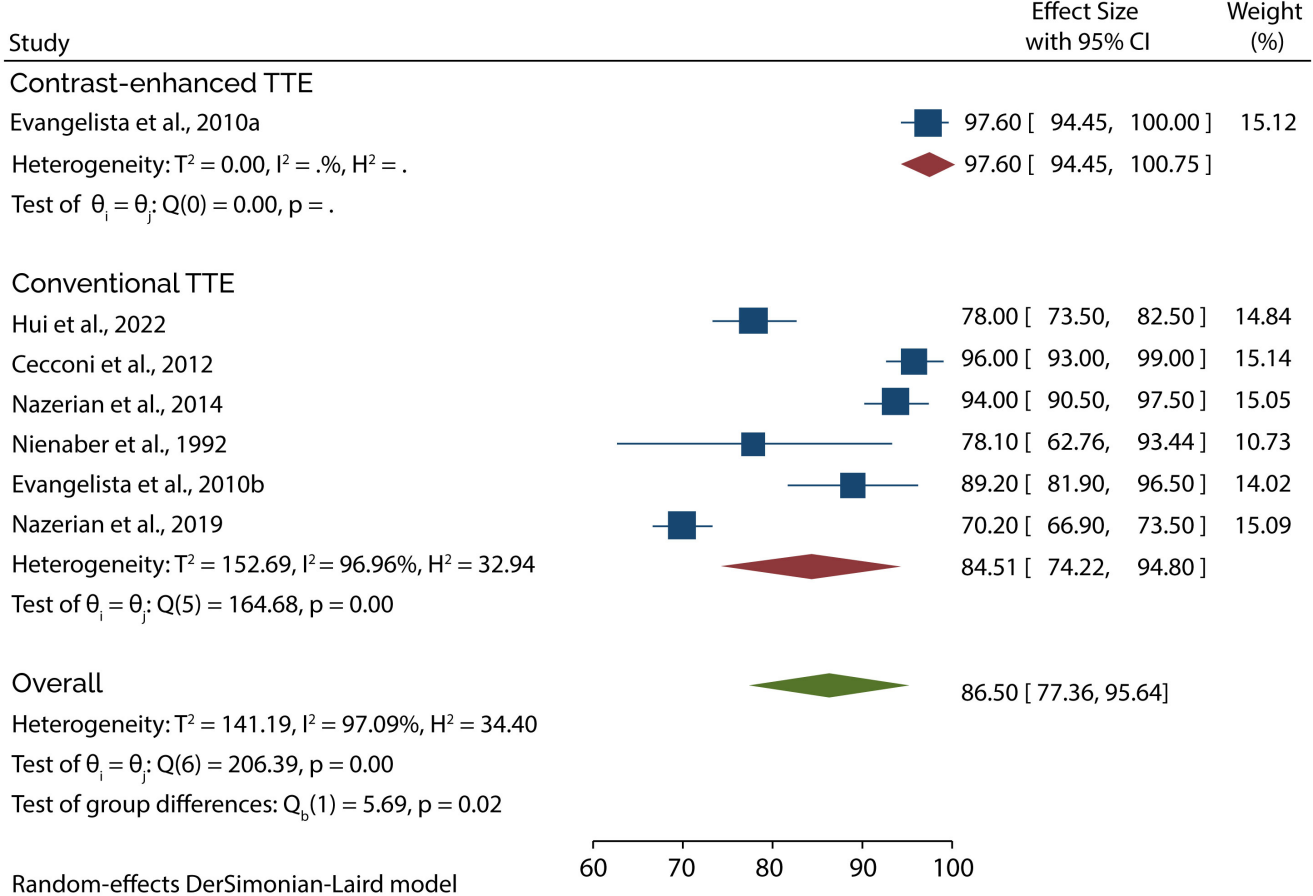
Forest plot of pooled specificity of TTE in diagnosing type A AAD.

Eight of the 11 studies evaluated the diagnostic role of TTE in type A AAD. A subgroup analysis of data from these studies showed that conventional TTE had a pooled sensitivity and specificity of 85.35% (95% CI: 78.40–92.30) (Figure 3) and 84.51% (95% CI: 74.22–94.80) (Figure 4), respectively. Conversely, subgroup analysis of data in one of the studies showed that contrast-enhanced TTE had a sensitivity and specificity of 93.30% (95% CI: 85.75–100) (Figure 3) and 97.60% (95% CI: 94.45–100) (Figure 4).

**Figure 5:**
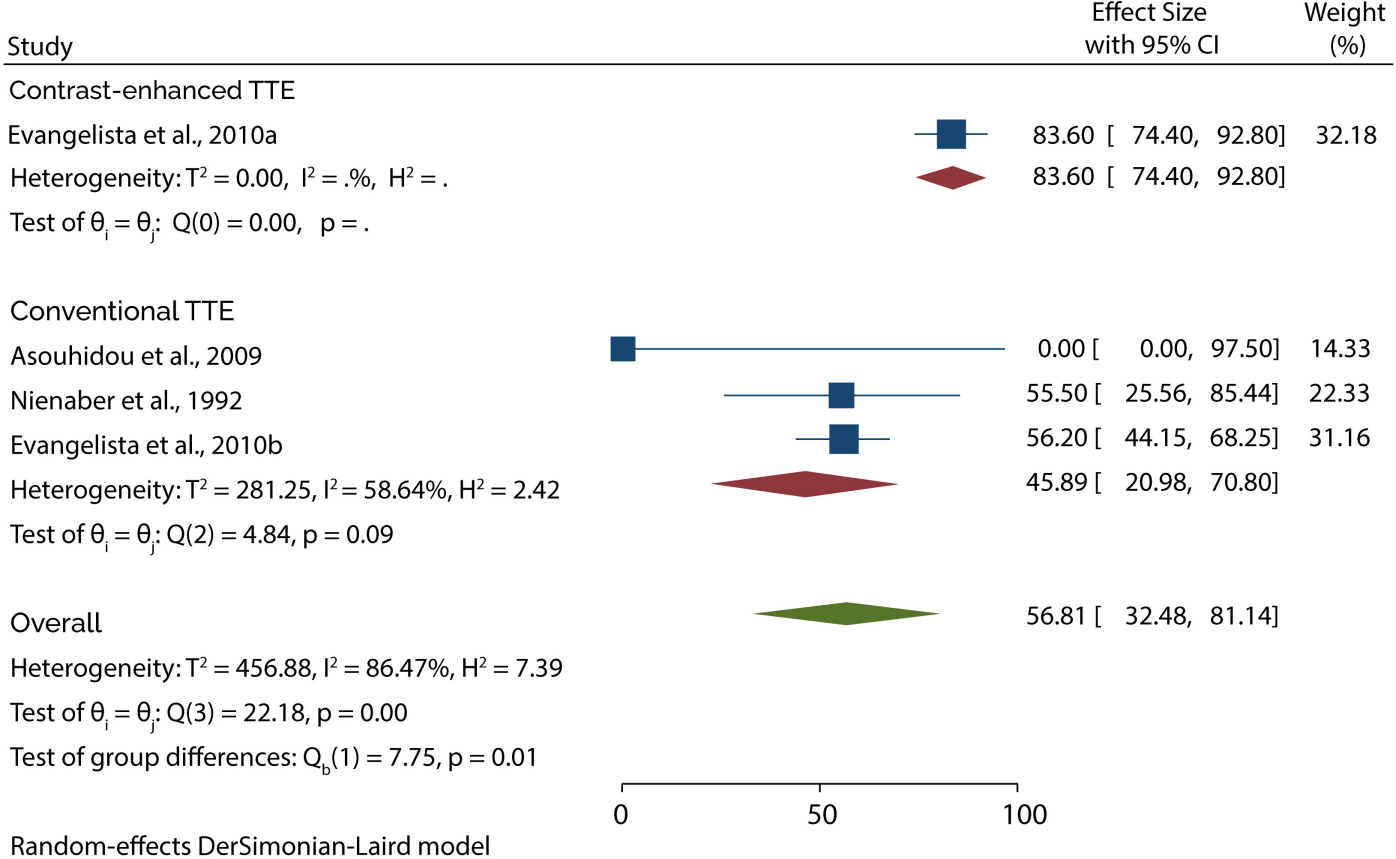
Forest plot of pooled sensitivity of TTE in diagnosing type B AAD.

**Figure 6:**
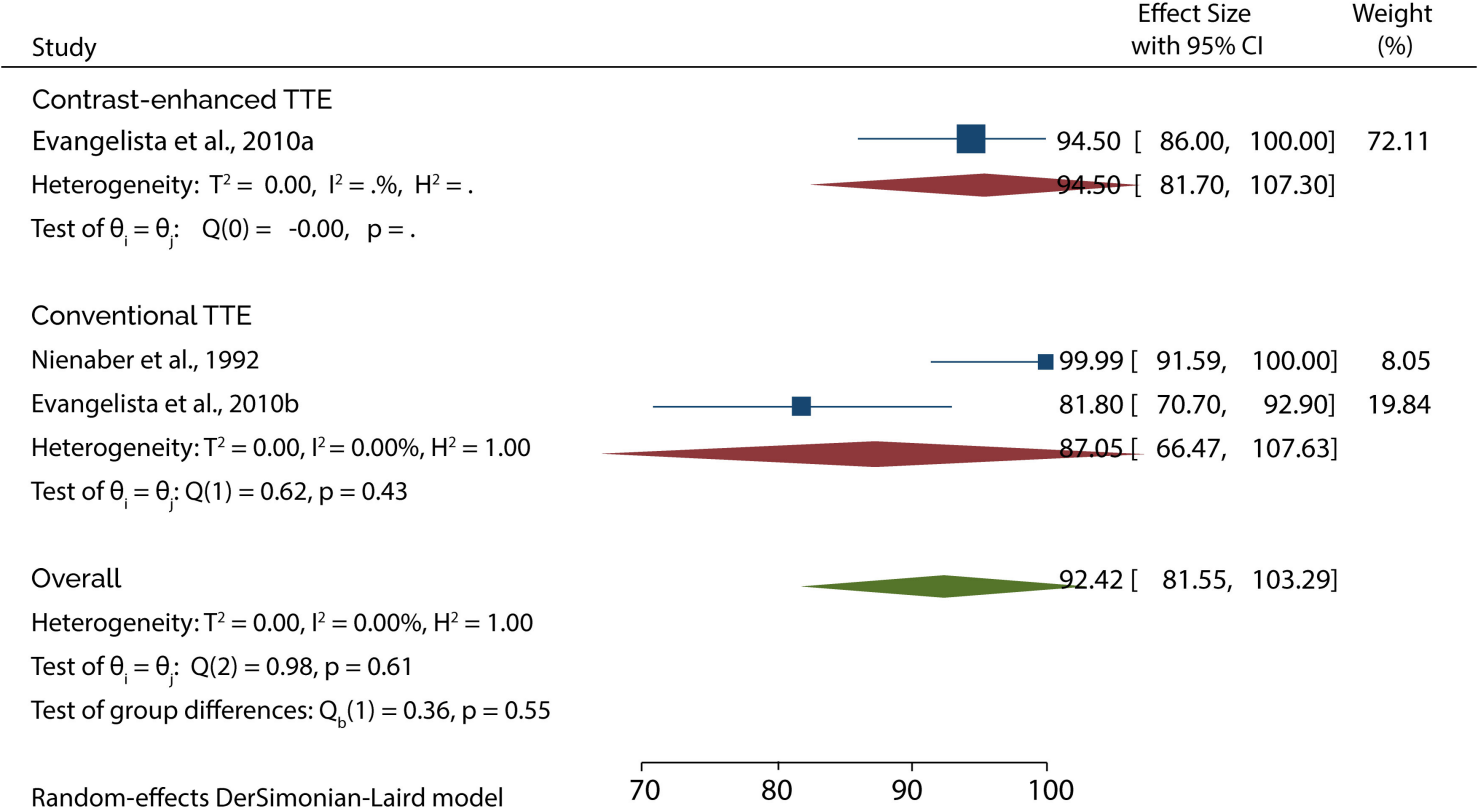
Forest plot of pooled specificity of TTE in diagnosing type B AAD.

On the other hand, 3 studies evaluated the role of TTE in diagnosing type B AAD. A subgroup analysis of data from these studies indicated that conventional TTE had pooled sensitivity and specificity of 45.89% (95% CI: 20.98–70.80) (Figure 5) and 87.05 (95% CI: 66.47–100) (Figure 6), respectively. In contrast, the subgroup analysis suggested that contrast-enhanced TTE had a sensitivity and specificity 83.60% (95% CI: 74.40–92.80) (Figure 5) and 94.50 (86–100) (Figure 6).

### TOE diagnostic accuracy

**Figure 7:**
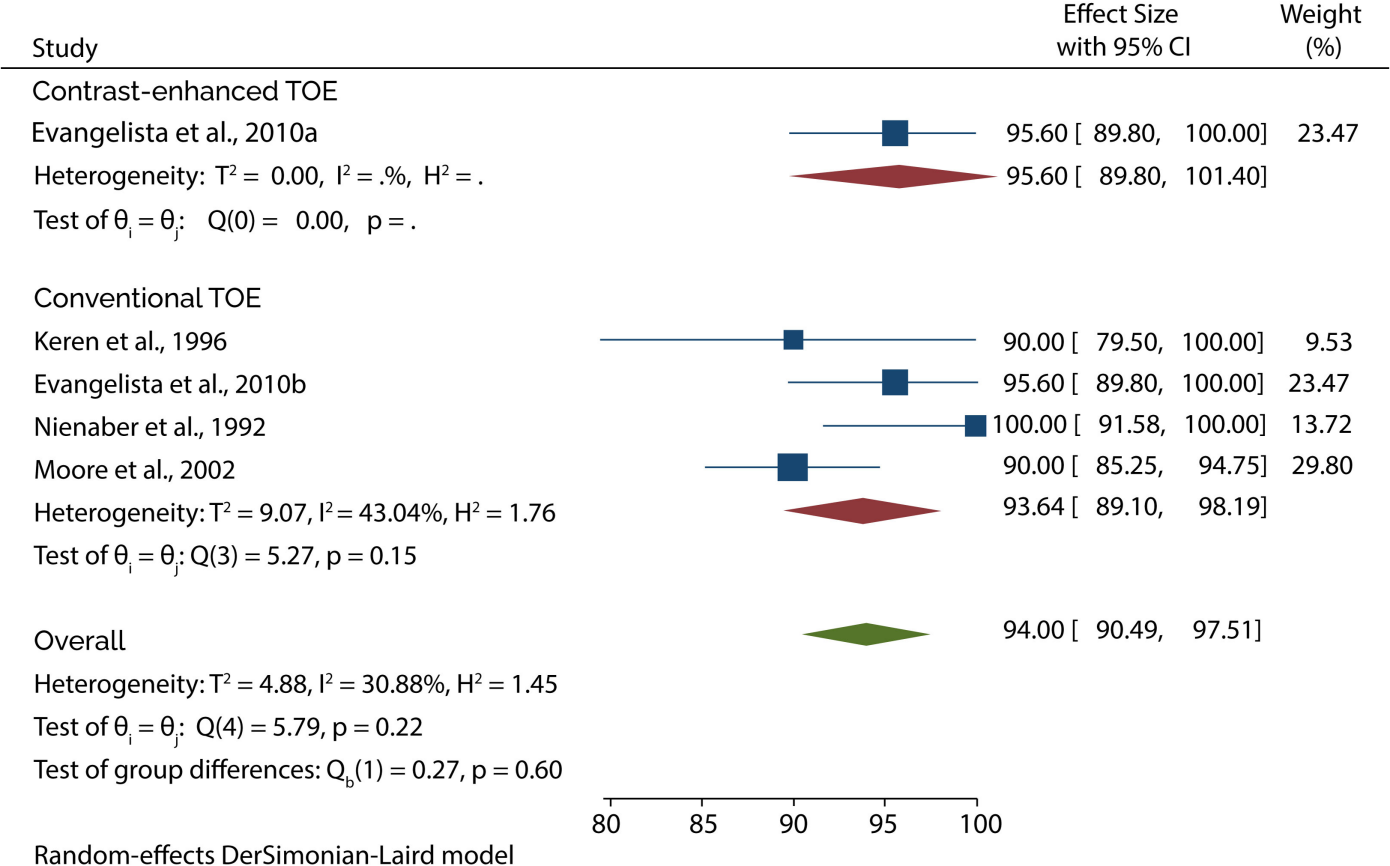
Forest plot of pooled sensitivity of TOE in diagnosing type A AAD.

**Figure 8:**
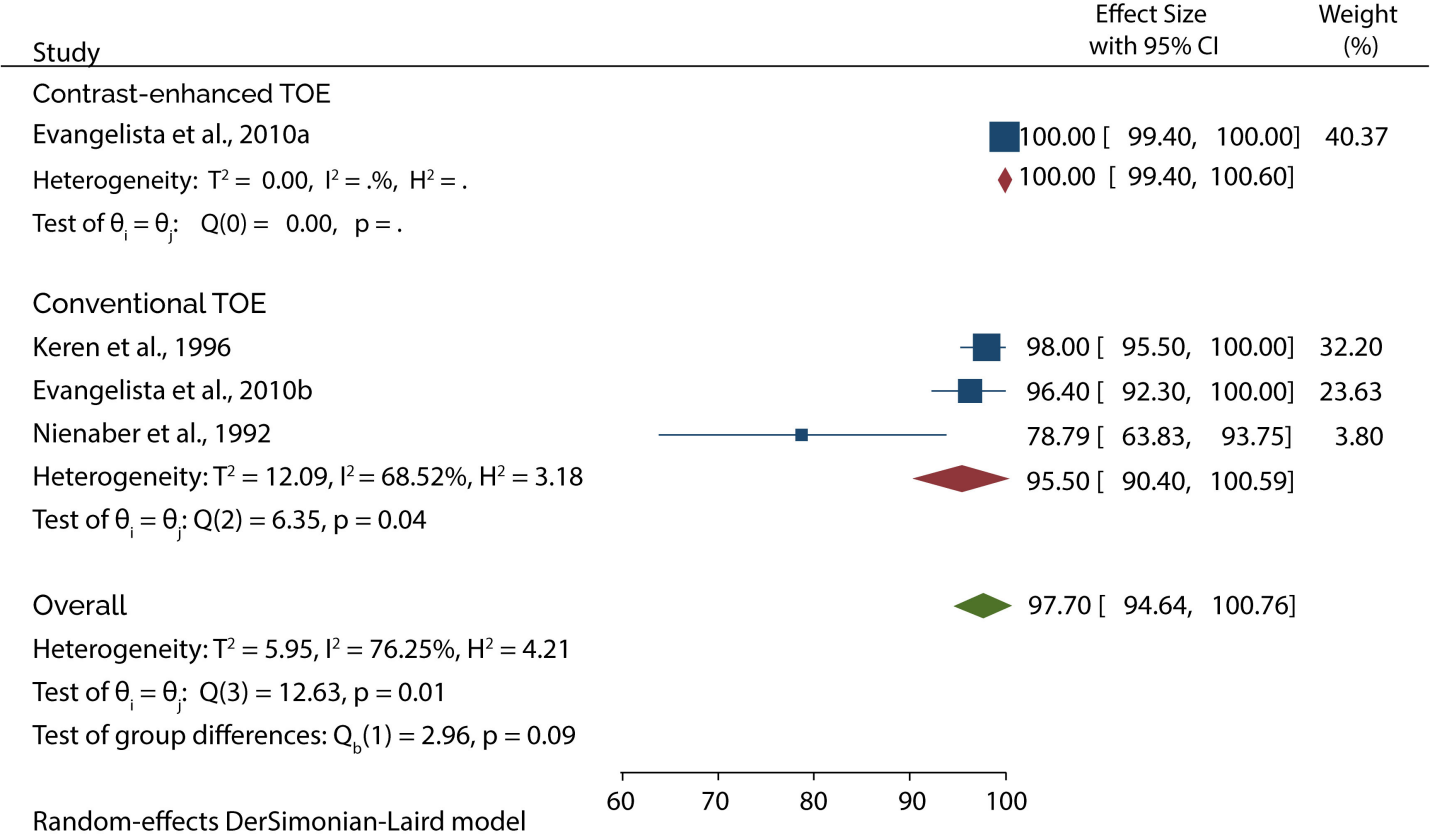
Forest plot of pooled specificity of TOE in diagnosing type A AAD.

The Diagnostic accuracy of TOE in diagnosing AAD was discussed in 5 included studies. Four out of 5 studies evaluated the diagnosis of type A AAD, of which the subgroup analysis showed that conventional TOE was 93.64% sensitive and 95.50% specific, while contrast-enhanced TOE was 95.60% sensitive and 100% specific (Figures 7, 8).

**Figure 9:**
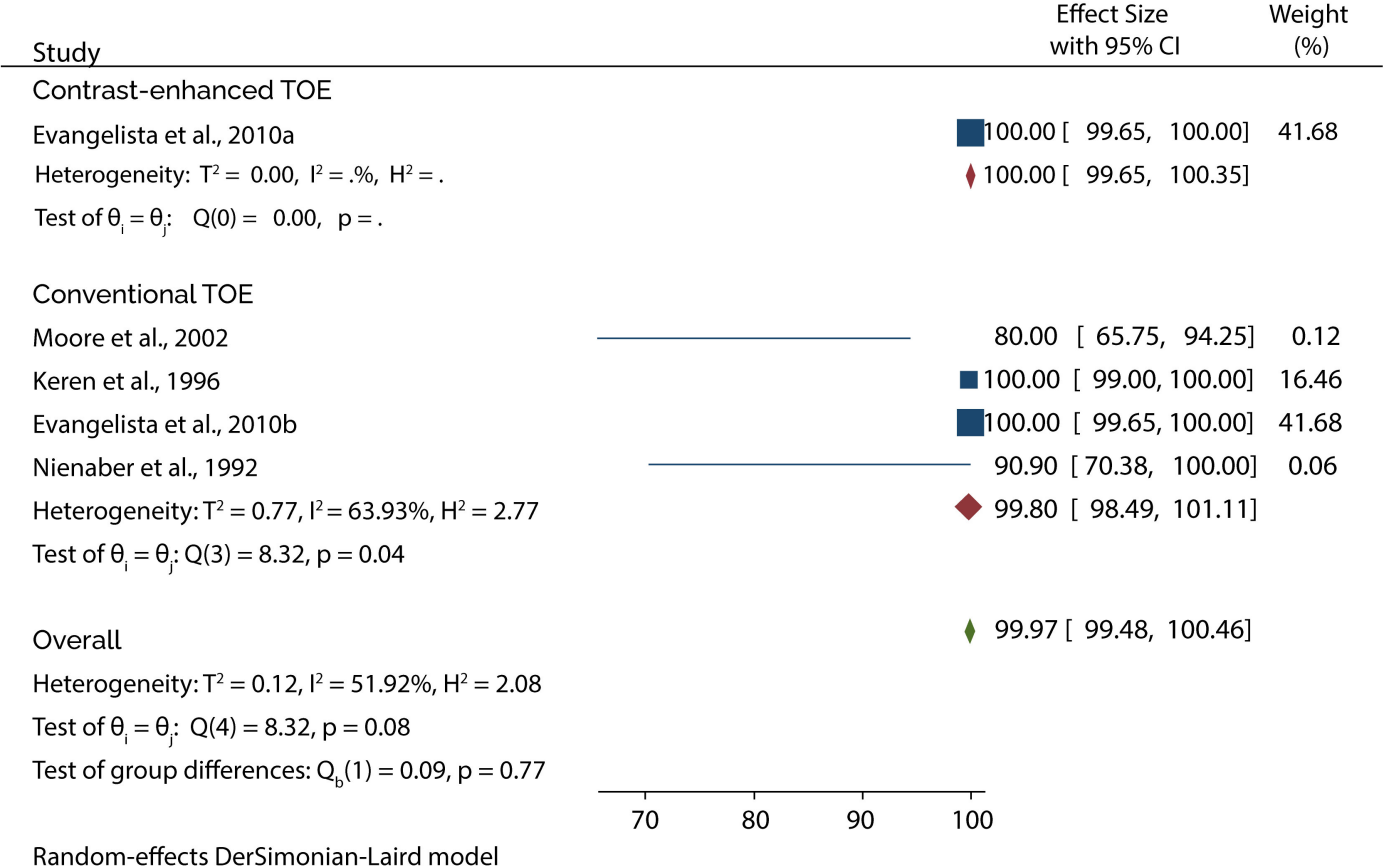
Forest plot of pooled sensitivity of TOE in diagnosing type B AAD.

**Figure 10:**
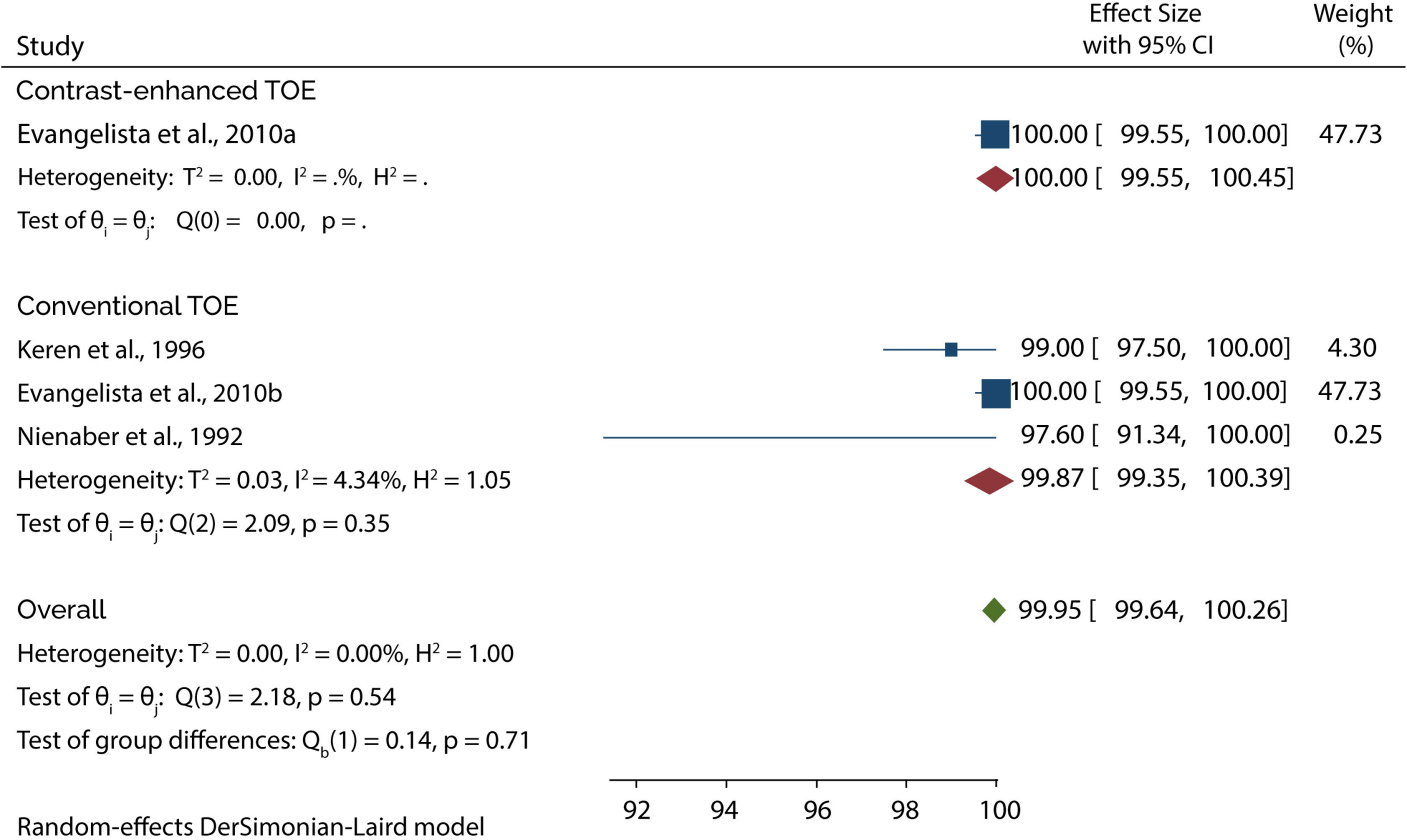
Forest plot of pooled specificity of TOE in diagnosing type B AAD.

On the other hand, 4 of the 5 studies evaluated the diagnosis of Type B AAD, of which the overall sensitivity and specificity of conventional TOE was 99.80% and 99.87%, while contrast-enhanced TOE was 100% sensitive and specific (Figures 9, 10).

### Misdiagnosis

The pooled data from studies evaluating the role of TTE in diagnosing AAD shows that false negatives occur at a rate of 28.6%, while false positives occur at a rate of 18.6%. On the other hand, the pooled data for studies evaluating TOE shows that false positives and negatives occur in about 2.4% and 4.3% of the patients undergoing this diagnostic criterion for AAD (Table 2).

**Table 2:** Pooled echocardiography false negatives and false positives.

Index test	Number of studies	Event rate	95% CI
TTE			
False negatives	4	0.286	0.087–0.629
False positives	4	0.186	0.051–0.492
TOE			
False negatives	3	0.024	0.11–0.049
False positives	4	0.043	0.016–0.111

## Discussion

AAD represents a medical emergency with a high mortality rate within the first few hours of symptom onset. Therefore, it is essential to have rapid and accurate imaging tools to diagnose this condition. In the present study, we have evaluated the role of TTE and TOE in the diagnosis of AAD and found that conventional TOE has a better sensitivity and specificity for the diagnosis of both Type A and type B AAD compared to conventional TTE. However, our subgroup analysis has shown that contrast-enhanced TTE results in similar diagnostic accuracy as the conventional TOE. Moreover, pooled results show that misdiagnosis is lower when TOE is used for AAD diagnosis than when TTE is used.

We have shown that TTE is inferior to TOE in diagnosing AAD. This finding aligns with a previous French multicenter study which recorded sensitivity and specificity of 55% and 89% for TTE and 96% and 94% for TOE. Furthermore, the study concluded that TOE is far superior to TTE when the thoracic descending aorta and the aortic arch are involved (20). These results are further reinforced by a previous meta-analysis which did not distinguish the data for AAD patients. That study showed that TOE had a sensitivity and specificity of 98% and 95%, respectively, and was comparable to other imaging techniques such as MRI and Helical CT (7). Even though our results have shown very high sensitivity and specificity for the TOE in diagnosing AAD, specificity as low as 78% and sensitivity of 80% have been recorded. However, this does not mean that the results of those studies are not accurate since they fall within the range outlined in the 2014 guidelines on the diagnosis and treatment of aortic diseases by the European society of cardiology (4).

It is worth noting that several variables can influence the sensitivity and specificity of TTE to match the accuracy of TOE. The first variable is the echocardiography level of training. In the study by Cecconi and colleagues, where the TTE was carried out by well-trained operators, the sensitivity and specificity for diagnosing classical type A AAD (CAAAD) was very high (90% and 96%, respectively) (9). Similarly, Sobczyk and Krzysztof reported that the echocardiographic examinations were performed by an experienced echocardiographer, and the reported sensitivity for AAD was very high (99.42%) (11). These results indicate that the TTE should be carried out by well-trained, experienced echocardiographers who can achieve optimal images and interpret them correctly to improve the diagnostic accuracy of this imaging technique in AAD. Evidence also shows that patient selection impacts the outcomes of TTE in diagnosing AAD. Sobczyk and Krzysztof reported that the sensitivity of TTE reached 99.4% when diagnosing type A AAD because the data was retrieved from patients transferred to their center with an already established suspicion of AD in about 50% of the patients (11). Moreover, with the recent technological advancements, harmonic imaging in TTE has been employed to improve diagnostic accuracy. In harmonic imaging TTE investigations, the frequency is generally double the fundamental frequency to decrease the artifactual echoes emanating from hazy tissue-tissue and tissue-blood interfaces. Thus, this approach offers a better echo graphic signal with an enhanced noise ratio and improved lateral resolution. Owing to this, the overall clarity of pictures is increased, and better identification of thin linear features like aortic intimal flaps or distinct wall thickening may be noticed (9, 21, 22).

In recent years contrast echocardiography has also been used to improve the accuracy of diagnosing AAD (23). Our subgroup analysis has shown that transthoracic contrast echocardiography significantly improved the accuracy of diagnosing AAD and produced similar outcomes as the conventional TOE. This finding is supported by a previous research article which reported that the specificity of conventional TTE increased from 73.68% to 100% after contrast enhancement (24). Moreover, that study found that contrast-enhanced TTE correctly diagnosed AAD in all patients, while the conventional TTE had 5 false positive results. In addition, our subgroup analysis has shown a slight improvement in the diagnosis of AAD when using contrast TOE as opposed to conventional TOE. Similarly, Agricola and colleagues found that the sensitivity and specificity of conventional TOE were slightly improved using contrast enhancement (91% and 95% vs. 100%, without and with contrast enhancement, respectively) (15).

Even with the superiority of TOE in diagnosing AAD, our results have shown that false negatives and positives are observed during the diagnosis. This shows that the AAD diagnosis cannot be excluded confidently based on a single TOE test finding. It is strongly recommended that additional imaging tests are performed if the initial diagnostic tests do not identify AD despite the diagnosis being clinically suspected. It is also essential to have other confirmatory imaging tests for patients diagnosed with AAD using the TOE test. Evidence from the study by Agricola and colleagues seems to suggest that contrast-enhanced TOE can eliminate the rate of false positives and negatives (15). According to this study, the conventional TOE had 2 false negatives and 2 false positives in diagnosing AAD, but no false results were recorded when contrast TOE was used. However, the results of this study cannot be used to guide clinical care since it included a very small sample with AAD. Additionally, the study excluded some patients with type A and B AAD for various reasons, including shock. Therefore, the study population was limited, meaning their results might have been influenced.

Apart from diagnosing AAD, echocardiography is also essential in diagnosing AD complications and other differential features which increase the risk of mortality in these patients. One of the most frequent complications which can be diagnosed and quantified using echocardiography is aortic regurgitation (AR) which occurs in 40%–76% of patients presenting with type AAD (25). TOE usually provides better information on the mechanisms of AR, which may influence the surgical decision for valve replacement or repair. Nienaber and colleagues reported that TOE and TTE have similar accuracy in diagnosing AR (100% sensitivity and 87.5 specificity for both echocardiography techniques). Further analysis in this study shows that TOE and TTE are even more sensitive than MRI in diagnosing AR; however, more trials should be carried out to establish this finding. Echocardiography is also critical in diagnosing arterial vessel involvement since visceral or peripheral mal perfusion syndrome is highly associated with increased morbidity and mortality. Evidence in the study by Evangelista et al. (13) showed that TTE was more helpful in diagnosing supra-aortic vessel involvement than TOE with and without contrast enhancement. However, it was reported that TOE made the correct diagnosis in all 19 patients with subclavian artery involvement. Further analysis showed that TTE was limited in diagnosing coronary artery and coeliac trunk involvements, while TOE with and without contrast enhancement was able to diagnose 4 patients with coronary dissections confirmed surgically and 19 dissections of coeliac trunk confirmed by CT. The study also showed that none of the echocardiographic techniques could diagnose lower vessel involvement.

Pericardial effusion is another chronic complication that arises in around 40% of individuals with type A acute aortic syndromes (AAS) (25). The existence of pericardial effusion could signal a rupture of the false lumen in the pericardium; however, it might be due to the adventitia irritation induced by aortic hematoma or a minor effusion from the wall. Evidence suggests that echocardiography is a good diagnostic tool for pericardial effusion and cardiac tamponade. For instance, Nienaber and colleagues reported that of the 53 patients with clinically suspected AAD, TTE correctly diagnosed pericardial effusion in 6 patients, while TOE diagnosed pericardial effusion in 7 patients (14). However, TOE was more sensitive than TTE when diagnosing the presence of pericardial effusion. Further analysis showed that the pericardial effusion was associated with type A dissection. This finding is reinforced by Hui and colleagues, who reported that TTE diagnosed pericardial effusion/cardiac tamponade in 48.76% of the patients presenting with type A AAD (17).

In addition to diagnosing AD complications, echocardiography is essential in identifying true and false lumen. The ability to distinguish between the true and false lumen is critical since placing an endoluminal stent graft in the false lumen can have hazardous consequences. However, the distinguishment between the two is not very straightforward, but the following features can be used; the first feature is about the size. Often the false lumen is characterized by a large-sized lumen due to the higher false luminal pressures (26, 27). The second feature is pulsation, of which the true lumen is characterized by systolic expansion while the false lumen is characterized by systolic compression (28). Thirdly, the flow direction can be used to distinguish between the two, of which the true lumen is characterized by systolic antegrade flow while the false lumen is characterized by reduced or absence of systolic antegrade flow or by a retrograde flow (29). Finally, the contrast echo flow in a true lumen is early and fast, while it is delayed and slow in a false lumen. Evidence shows that TTE and TOE are good imaging techniques for identifying false and true lumen. Evangelista and colleagues reported that TOE better visualized the false lumen entry tear than TTE (*p* < 0.001). However, the conventional TOE could not correctly identify the true lumen in 6 patients (8.8%) (13). In all these cases, the intima was immobile in the M-mode, and the color doppler showed that both lumina had a similar flow pattern. When the contrast-enhanced TOE was applied, all six cases were correctly identified as the true lumen. Further analysis showed that the conventional TTE and TOE could not identify the false lumen flow direction; however, the contrast-enhanced TOE correctly diagnosed 9 cases of false lumen while TTE correctly diagnosed false lumen in 7 patients.

### Limitations

Like any other scientific research study, our review was limited in some aspects. First, we only included studies published in English, meaning that some of the relevant studies that would have been used in the analysis were excluded, therefore, introducing a selection bias in our study. Secondly, we have a substantial heterogeneity in some of the analyses; however, the heterogeneity did not influence the results of our meta-analyses since most of the studies were of good methodological quality. Thirdly, the studies included in this review did not have one specific reference standard, which might have resulted in the high heterogeneity and influenced the diagnostic accuracy of the index test since not all reference standards provide an accurate diagnosis. Moreover, the studies included in this review are few due to the strict eligibility criteria, which did not include studies that discussed AD but did not differentiate the results for patients with the acute phase. Finally, one study in this review only included patients referred to the medical center, with 50% of the patients already having been diagnosed with AAD; this might have influenced the diagnostic accuracy reported in that study and subsequently influenced our results (11).

## Conclusion

TOE is a favorable diagnostic test with excellent sensitivity and specificity for detecting AAD. However, false positives and negatives are still a problem, meaning that additional imaging tests are required to make a correct diagnosis. Our subgroup analysis also shows that contrast-enhanced TTE can produce similar results to conventional TOE in diagnosing AAD. Therefore, in emergency settings where TOE is not readily available, this imaging modality can be used for the diagnosis. However, it should be noted that both TTE and TOE should be performed by experienced and well-trained operators and interpreters for the correct diagnosis of AAD.
